# Functional Ultrasound Localization Microscopy on Freely Moving Rats

**DOI:** 10.21203/rs.3.rs-9953849/v1

**Published:** 2026-06-10

**Authors:** Yike Wang, Bohan Zhao, Matthew R. Lowerison, Bing-Ze Lin, Zhe Huang, YiRang Shin, Ani Hovhannisian, Laura Bohn, Li Ye, Pengfei Song

**Affiliations:** Duke University; Scripps Research; Duke University; Duke University; Duke University; Duke University; Scripps Research; University of South Florida; Scripps Research; Duke University

## Abstract

Brain-wide functional neuroimaging at single-vessel resolution in naturally behaving animals has been challenging to realize. Functional ultrasound localization microscopy (fULM) offers brain-wide hemodynamic imaging at microscopic resolution but has remained restricted to head-fixed preparations. Here we present a platform for fULM in freely moving rats. The system integrates a magnetic probe-clamping interface, a counterbalanced pulley-based tether, chronic jugular venous access for on-demand microbubble delivery, and a motion-correction processing pipeline tailored to freely moving ULM data. Using visual stimulation, we first show that the platform reliably captures functional hemodynamic responses and supports super-resolved vascular reconstruction across deep brain regions. We then demonstrate that freely moving fULM resolves stimulus-evoked vessel-specific changes in both diameter and flow velocity, revealing microvascular heterogeneity that cannot be recovered with conventional methods. Finally, we apply the platform to image brain responses induced by an experimental biased μ opioid agonist (SR-17018) in rats, revealing transient, region-dependent cerebrovascular responses, with flow-velocity changes of 20–30% that vary in magnitude and temporal profile across individual vessel segments in cortex and periaqueductal gray in free-moving rats. This work establishes freely moving fULM as a practical platform for whole-brain microvascular imaging under naturalistic behavioral conditions.

## Introduction

One of the longstanding goals of functional neuroimaging is to map brain-wide activity with high spatiotemporal resolution in conscious subjects under naturalistic conditions^1,2^. Many central questions in systems neuroscience concern how distributed circuits coordinate activity across brain regions during naturalistic behavior^3^, from spatial navigation and memory consolidation to social interaction and reward-guided decision-making. Capturing these processes at the circuit level demands imaging tools that combine brain-wide coverage with sufficiently high spatial resolution in naturally behaving subjects^4^. Achieving this goal, however, requires a balance among three key imaging attributes: a large field of view (FOV) for unbiased whole-brain monitoring, high spatial resolution to resolve local microcircuits, and compatibility with naturally behaving subjects. The last requirement is particularly challenging in small-animal studies, where anesthesia is commonly used to suppress motion but introduces well-known confounds in neural activity and neurovascular coupling^5–7^.

Simultaneously achieving these requirements remains challenging, and existing imaging modalities satisfy only subsets of these criteria. Magnetic resonance imaging (MRI)^8^, for example, provides whole-brain coverage but confines subjects to rigid scanner environments that typically require sedation or mechanical immobilization of the animal^9^. Miniaturized optical microscopes enable imaging in freely moving rodents^10,11^, but their limited field of view captures only a small fraction of the brain, precluding the systems-level perspective that both circuit neuroscience and pharmacological studies demand^12^. Functional ultrasound imaging (fUS) has emerged as a compelling candidate to bridge this gap^13^. It has demonstrated compatibility with freely moving animals^1,2^, as well as in reward-guided behavioral studies^14^ and pharmacological neuroimaging^15,16^. However, its spatial resolution remains constrained by the acoustic diffraction limit (~ 100 μm)^13^, which is insufficient to resolve microvascular dynamics associated with local circuit activity.

Functional ultrasound localization microscopy (fULM) overcomes this resolution barrier^17^. By localizing intravenously administered microbubbles (MBs), based on ultrasound localization microscopy (ULM)^18–21^, fULM improves the spatial resolution of conventional fUS by nearly an order of magnitude (~ 10 μm) while retaining its penetration depth and coverage^22,23^. Recent studies have also further demonstrated the feasibility of fULM in head-fixed awake rodents^24^. In principle, fULM is uniquely positioned to deliver brain-wide, microscale hemodynamic imaging during natural behavior. However, the technique has not yet been extended to non-head-fixed conditions. The main obstacle is that fULM requires the continuous presence of microbubbles in the bloodstream, which in turn demands reliable intravenous delivery and stable MB concentrations in freely moving animals.

In this study, we establish a platform that extends fULM to freely moving rats, enabling brain-wide, super-resolution hemodynamic imaging during unconstrained naturalistic behavior. The platform integrates a chronically implanted jugular vein catheter for sustained microbubble delivery, a pulley-based tethering system for unrestricted movement, and a motion-robust fULM processing pipeline, collectively removing the practical barriers that have confined fULM imaging to head-fixed or anesthetized preparations. We validate the platform through a visual stimulation paradigm and demonstrate its reproducibility in capturing microvascular responses. To demonstrate the broader utility of this platform, we next apply it to pharmacological neuroimaging by mapping brain-wide microvascular responses to a subcutaneously administered opioid analgesic. Performing such measurements in naturally behaving animals is essential, as anesthesia and physical immobilization confound the hemodynamic signals of interest^5^. This caveat is particularly important when we study *in vivo* actions of analgesic agents such as the opioids. Using SR-17018, an experimental biased opioid agonist in animal models, as a proof-of-concept agent, we resolved drug-induced hemodynamic changes at the single-vessel level across the brain. This work expands the experimental space of functional neuroimaging by extending super-resolution hemodynamic imaging to freely behaving animals. It opens a new avenue for interrogating microvascular dynamics in behavioral and pharmacological contexts that were previously inaccessible.

## Results

### A freely moving fULM platform integrating stable probe fixation, chronic venous access, and counterbalanced tethering

**Figure 1:**
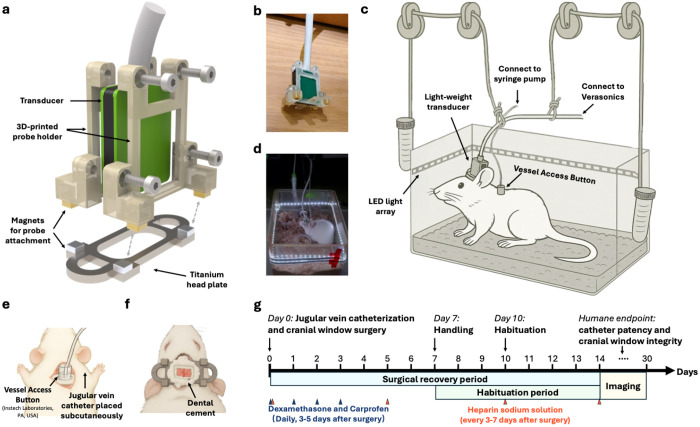
Experimental setup and procedure. **a,** Design of the probe fixation. The probe is secured using two 3D-printed holders, restricting movement in all directions. Magnets at the base of the holders enable secure attachment to a titanium head plate on the skull. **b,** Photo of the probe clamped by the holders. **c,**Schematic of the fULM imaging setup for a freely moving rat. The probe cable and intravenous catheter are suspended using a pulley-based counterbalanced tethered system, allowing unrestricted movement in the cage. An LED array surrounding the cage delivers visual stimulation. **d,** Photo of the setup during imaging. **e,** Illustration of jugular vein catheterization surgery. After catheter insertion into the jugular vein, it is tunneled subcutaneously to the back and connected to a Vessel Access Button, which allows repeated intravenous access during free movement. **f,** Illustration of the cranial window surgery. A titanium head plate is fixed to the skull using dental cement. An ultrasound window is created by removing part of the skull. Magnets on the head plate enable attachment of the imaging probe. **g,** Timeline of the experimental procedure. The full experiment includes surgeries, post-operative recovery, pre-imaging training, handling and habituation, and imaging sessions.

To perform fULM in freely moving rats, we developed a tethered imaging platform that addresses several engineering requirements: stable probe fixation that minimizes relative motion between skull and probe, rapid and precise probe attachment to the targeted imaging plane across experiment sessions, continuous and stable microbubble infusion without restricting animal movement, and counterbalancing of the probe cable weight during locomotion (Fig. 1a–d).

For probe fixation, a lightweight linear array probe was secured by two 3D-printed holders that clamp the probe bilaterally, constraining probe movement in all directions (Fig. 1a–b). The holders attach magnetically to the titanium head plate, with four magnets on the holder base that engages with four matching magnets bonded at predefined locations on the head plate surface. This magnet-to-magnet alignment allows the probe to be precisely positioned to the same imaging plane upon each attachment, which enables rapid mounting and removal throughout the imaging sessions.

Since the clamping design locks the probe position relative to the head plate, the imaging plane is fixed for a given holder after head plate implantation. To enable imaging plane selection without compromising the fixation stability, we fabricated a set of interchangeable holder pieces whose centerlines are offset from the probe midline by −1.5 to 1.5 mm in 0.3 mm increments, so that each offset produces a distinct imaging plane (Supplementary Figure 1). The full offset range covers a 3 mm cranial window along the anterior-posterior axis of the animal brain. The offset range and step size can be tailored to specific experimental needs based on cranial window size, 3D printing precision, and the elevational beam width of the probe. For a given animal, the imaging plane corresponding to each holder only needs to be pre-characterized once under anesthesia. In subsequent freely moving imaging sessions, the appropriate holder can be selected based on target-specific brain regions.

For continuous microbubble delivery, a jugular vein catheter was implanted and tunneled subcutaneously to an exteriorization point on the back, where it connects to an external syringe pump via the infusion line. The infusion line and probe cable were bundled together and routed by an overhead pulley, with a water-filled centrifuge tube on the opposing end serving as a counterweight (Fig. 1c–d). Counterbalance tension was adjusted by adding or removing water inside the tube. Under this configuration, animals moved freely within the cage (Supplementary Video 1). An LED array surrounding the cage delivered patterned visual stimulation for the functional imaging experiments conducted in this study.

Each animal underwent jugular vein catheterization (Fig. 1e) and cranial window surgery with head plate implantation (Fig. 1f), which were performed on the same day (see Methods for detailed protocols). Following surgery, animals were allowed a minimum of one week of recovery with post-operative analgesic and anti-inflammatory treatment before any experimental handling (Fig. 1g). Beginning on day 7, animals were gradually introduced to handling sessions to reduce anxiety, followed by habituation to the imaging setup from day 10, during which counterbalance effectiveness was monitored to ensure minimal head loading. Imaging sessions commenced after the full two-week recovery and habituation period. With proper post-operative care, all animals in this study survived beyond four weeks after surgery. Humane endpoints were defined by loss of catheter patency or deterioration of cranial window integrity, at which point the animal was euthanized.

### Functional validation and motion-corrected super-resolution reconstruction establish the prerequisites for freely moving fULM

**Figure 2:**
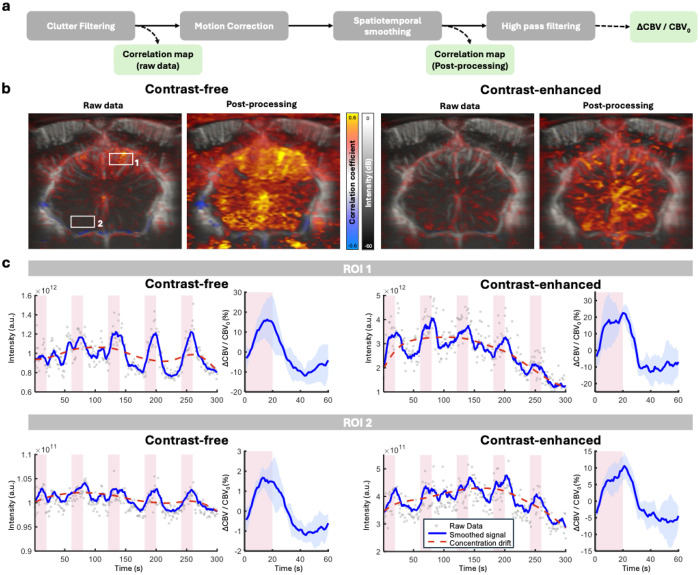
Functional ultrasound imaging on the freely moving rat with and without contrast injection. **a,** Workflow for data processing. Steps include clutter filtering, motion correction, spatiotemporal smoothing, and high-pass filtering. The final hemodynamic response is expressed as the relative change in cerebral blood volume (ΔCBV/CBV_0_). **b,** Representative fUS imagesduring visual stimulation with and without microbubble contrast. For each condition, power Doppler (grayscale) images are overlaid with correlation maps. Both the raw blood flow signal obtained directly after clutter filtering and the signal after post-processing are used for correlation analysis. White boxes denote two regions of interest (ROIs) selected for time course extraction. **c,** ROI-based time courses for contrast-free (left) and contrast-enhanced (right) fUS. Grey dots show raw signal intensity, blue lines indicate smoothed signals, and pink shaded areas represent stimulation epochs. In the contrast-enhanced case, slow microbubble concentration drifts are modeled (red dashed line) and removed by high-pass filtering. Right subpanels show cycle-averaged hemodynamic responses expressed as ΔCBV/CBV_0_ (mean ± variability shading).

To validate the functional imaging capability of the platform, visual stimulation was applied under both contrast-free and contrast-enhanced conditions. The resulting hemodynamic responses were quantified as correlation maps and ΔCBV/CBV_0_ (Fig. 2a–b; see Methods for details). In both conditions, the superior colliculus (ROI 1), a primary visual processing region, exhibited a clear stimulus-locked response with a peak ΔCBV/CBV_0_ of approximately 20% and a return to baseline within 15-20 s after stimulus offset (Fig. 2b–c). These response characteristics are consistent with existing studies under head-fixed awake imaging configurations^3^.

One practical consideration with contrast-enhanced imaging is that microbubble concentration inevitably changes over time during freely moving recordings, even with our catheterization setup. Over the 5-min acquisition window, signal amplitude decreased by approximately 30-40% (Fig. 2c, ROI1, red dashed line). When the drift remains moderate, however, it can be effectively corrected using high-pass filtering (cutoff ~0.01 Hz), a standard approach in the functional ultrasound processing (also known as detrending^23,25^). In our data, stimulus-evoked responses remained clearly detectable through the final stimulation cycle. On the other hand, contrast-enhanced imaging provides clear sensitivity gains for deep brain structures. For example, in the substantia nigra (ROI 2), the contrast-free condition yielded only a weak response of approximately 2%, as ultrasound attenuation at depth reduces blood scattering intensity and limits the detection of small vessels. With microbubble contrast, the response amplitude increased to approximately 10% (Fig. 2b–c).

Beyond the sensitivity gains described above, contrast-enhanced acquisition enables fULM processing for super-resolution imaging. While fUS reliably captures whole-brain hemodynamic responses, its spatial resolution (~100 μm) remains insufficient to resolve individual vessels. We therefore performed fULM reconstruction using the contrast-enhanced dataset.

**Figure 3:**
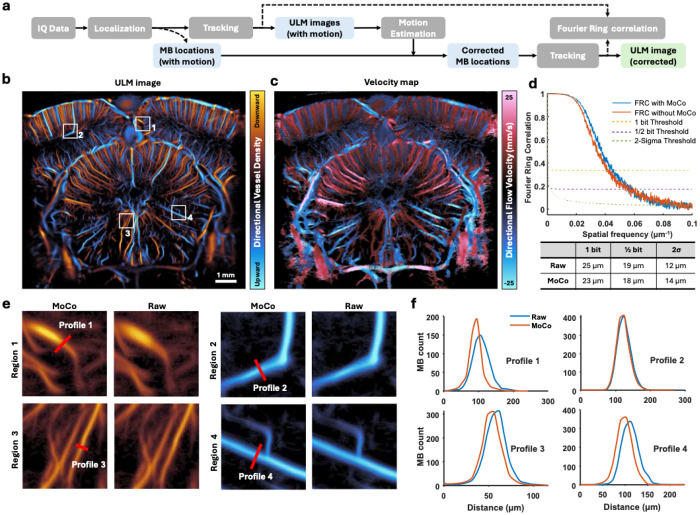
High-resolution ULM imaging in freely moving rats and comparison before and after motion correction. **a,** Workflow for data processing and motion correction. After microbubble (MB) localization and tracking, the motion field is estimated for each 10-second ensemble. This estimated motion field is applied to correct MB positions before re-tracking. Resolution of the ULM images before and after correction is assessed via Fourier ring correlation (FRC) analysis. **b,** ULM directional vessel density map after motion correction. Four ROIs are selected for zoom-in inspection. **c,** Corresponding directional flow velocity map. Flow direction is defined with downward flow as positive. **d,** FRC curves comparing raw and motion-corrected datasets. Estimated resolution values under different thresholds (1-bit, ½-bit, 2σ) are summarized in the table. **e,** ROI-specific comparison of vascular structure before and after motion correction. **f,** Intensity profiles of microbubble (MB) counts along red lines in each ROI in **e**.

In this study, we observed internal brain tissue motion during freely-moving behavior, which manifested as slow positional drift that oscillated over extended recordings (see Supplementary Video 2). To ensure ULM imaging fidelity, we incorporated a novel motion correction step into the ULM pipeline (Fig. 3a). For each short temporal ensemble (10 s), a non-rigid deformation field was estimated from the ULM images using the ANTs (Advanced Normalization Tools) framework^26,27^ (see Methods for details). The estimated motion field was then applied to the localized microbubble coordinates, and the tracking step was re-executed on the corrected coordinates, which producing the corrected vascular density and velocity maps (Fig. 3b–c). By operating at the coordinate level rather than on the final reconstructed images, this motion correction strategy avoids velocity calculation errors in tracking, thereby preserving the accuracy of velocity quantification in ULM. The effectiveness of the motion correction is also illustrated in Supplementary Video 2.

Fourier ring correlation (FRC) analysis confirmed the benefit of motion correction on spatial resolution (Fig. 3d). In the 0.02-0.06 μm^−1^ frequency range, corresponding to vessel-scale features of tens of micrometers, the post-correction FRC curve consistently exceeded the pre-correction curve, indicating improved reconstruction fidelity at these spatial scales. ROI-level comparisons (Fig. 3e–f) revealed spatial heterogeneity in the correction effect. In superficial cortical regions that were most affected by motion (Region 1), vessel profile width narrowed from approximately 200 μm to 150 μm with sharper boundaries, whereas Region 2, which had minimal motion, showed negligible change. In deeper regions (Regions 3 and 4), where motion was predominantly translational, correction primarily improved vessel positional alignment. Together, these results indicate that the probe fixation strategy alone already provides sufficient stability for ULM to resolve cerebrovascular architecture during free movement. The motion correction further sharpens single-vessel definition, providing the spatial precision necessary for single-vessel-level functional analysis.

### Freely moving fULM resolves stimulus-evoked microvascular dynamics at single-vessel resolution

**Figure 4:**
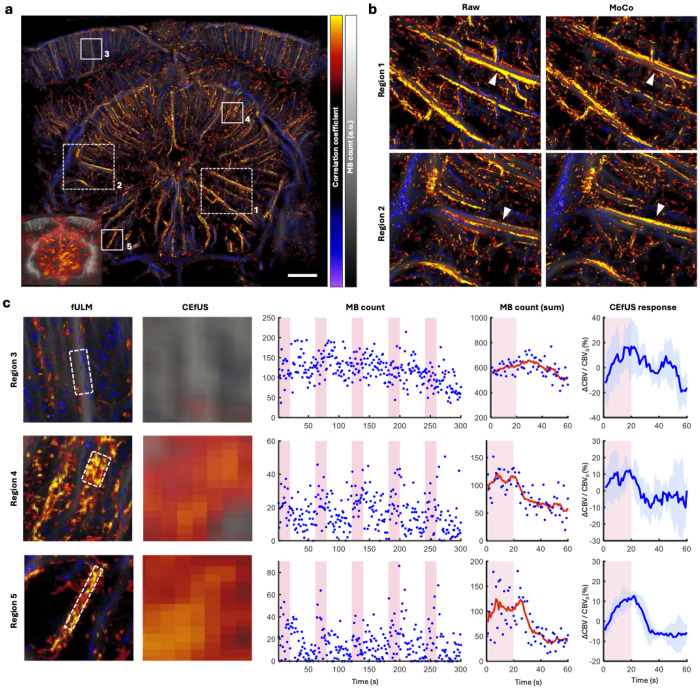
Functional correlation analysis of ULM in response to the visual stimulation. **a,** Correlation map derived from fULM superimposed on a grayscale structural ULM image. A contrast-enhanced fUS (CEfUS) correlation map from Figure 2 is shown in the lower-left corner for comparison. **b,** Enlarged views of Regions 1 and 2 (dashed boxes in a) before and after motion correction. These regions reveal that motion correction significantly changes the localized vascular responses in some vessels (arrowheads). **c,** Comparison of fULM and CEfUS responses in three selected ROIs (solid square boxes in a). For each selected region, fULM correlation maps (left) and corresponding CEfUS maps (right) are shown. Time courses of MB count in individual vessel segments are plotted across five stimulation cycles (pink-shaded epochs), along with MB count trends (red lines) (five-cycle summation) and CEfUS-derived ΔCBV responses (blue lines). fULM reveals spatially confined functional responses with superior resolution compared to CEfUS.

To evaluate functional imaging performance under freely moving conditions, functional correlation maps based on MB counts were computed and overlaid on the structural ULM images (Fig. 4a). Compared with the contrast-enhanced fUS (CEfUS) correlation maps derived from the same dataset, fULM showed a highly consistent spatial distribution of activation, with signals localized to the superior colliculus and deeper midbrain regions, but resolved functional responses at the level of individual vessels rather than the region-level activation patterns observed in CEfUS.

The effect of motion correction on the functional maps was evaluated in two representative regions (Fig. 4b). In uncorrected data, several vessels (arrowheads) showed strong positive correlation immediately adjacent to strong negative correlation, a characteristic signature of motion artifact in which stimulation-evoked tissue displacement shifts vessel positions into neighboring pixels, producing spurious correlation patterns. After correction, positive correlation appeared on both sides of the same vessel, as expected when vessel dilation during stimulation increases the microbubble count in pixels of vessel wall. These results indicate that motion correction effectively removed motion bias and improved the reliability of the functional maps.

To compare the spatial resolution of fULM and CEfUS, functional responses were examined in three representative ROIs (Fig. 4c). In a cortical ROI (Region 3), neither method detected a stimulus-related response. In two activated ROIs (Regions 4 and 5), CEfUS showed only spatially unresolved activation, whereas fULM localized responses to individual vessels whose microbubble count time courses closely followed the CEfUS ΔCBV temporal profiles. The single-vessel resolution of fULM further enabled quantification of flow velocity within individual vessel segments, which showed a similarly high correlation with the stimulation pattern.

**Figure 5:**
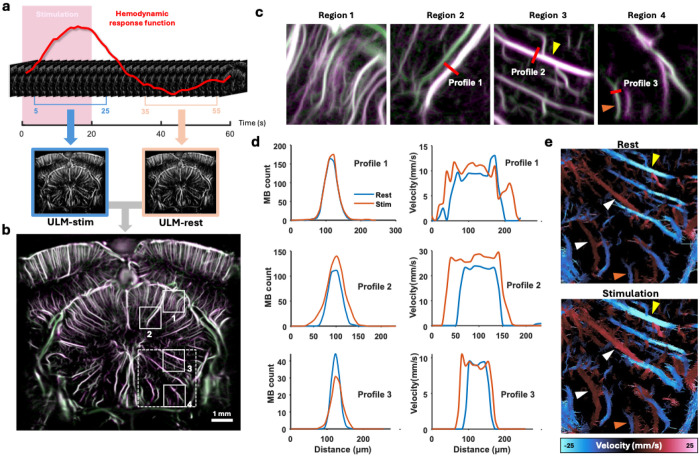
ULM-based mapping of vascular structural and velocity changes during visual stimulation. **a,** ULM frame selection based on hemodynamic response. A hemodynamic response function was extracted from Figure 2. ULM data were accumulated during the response peak (5–25 s, stimulation) and during a time-matched baseline period (35–55 s, rest) across five trials. **b,** Differential overlay of ULM images from stimulation and baseline periods (white, shared structures; green, baseline-dominant; purple, stimulation-dominant). **c,** Enlarged views from four ROIs (indicated by the solid square boxes in **b**) highlighting local vascular changes. **d,** Vessel profiles extracted from three ROIs in (c).Individual vessels exhibit distinct responses, including dilation and increased flow velocity during stimulation compared to rest. **e,** Velocity maps from the dashed boxed region in (b) highlighting stimulation-induced flow and diameter changes. Velocity map illustrates concurrent increases in vessel diameter and flow speed. Yellow and orange arrows indicate responsive vessels in Regions 3 and 4, respectively; white arrows mark additional vessels with clear stimulation-dependent differences.

To quantify stimulus-evoked responses at the single-vessel level, two time windows were defined within each 60-s stimulation cycle on the basis of the hemodynamic response: a stimulus-peak window (5-25 s) and a time-matched resting window (35-55 s) (Fig. 5a). By accumulating data across five cycles, approximately 100 s of effective acquisition were obtained per condition. The differential overlay of the two state-specific ULM images (Fig. 5b) reveals microvascular features preferentially present during stimulation (purple) or rest (green), with shared structures shown in white. In the four enlarged regions of interest (Fig. 5c), stimulus-associated microvascular features were identified in Region 1, located in the superior colliculus, as well as in Regions 2-4 in deeper midbrain areas.

Vessel diameter and flow velocity were compared between stimulation and rest for three profiles extracted from these regions (Fig. 5d). Profile 1 showed no appreciable change in either parameter. Profile 2 exhibited concurrent increases in both diameter and velocity. Profile 3 showed primarily vessel dilation, with limited velocity change. These patterns suggest that visually evoked flow regulation operates through vessel-specific combinations of diameter and velocity modulation, a level of heterogeneity that can only be resolved when both parameters are captured simultaneously across a large field of view.

Changes in vessel diameter between stimulation and rest can be further visualized through the velocity maps (Fig. 5e), because they are more sensitive to microbubble variations at vessel boundaries, and therefore reflect diameter changes more clearly. In the vessel indicated by the white arrow, both a velocity increase and vessel dilation consistent with Profile 2 were directly observed. Similar coupled changes in diameter and velocity were found at additional marked locations. Together, these results demonstrate that fULM in freely moving rats can stably resolve stimulus-evoked hemodynamic changes at the single-vessel level, revealing functional heterogeneity inaccessible to conventional power Doppler imaging.

### Freely moving fULM extends to pharmacological neuroimaging of SR-17018-induced cerebrovascular responses

With single-vessel flow-velocity quantification established under sensory stimulation (Fig. 5), this analytical framework was next extended to pharmacological neuroimaging. A key advantage of enabling fULM in free-moving animals (vs. anesthetized animals) for pharmacological imaging is that it avoids potential drug interactions with anesthetics during imaging. This ability is particularly important for studying *in vivo* brain responses to anesthetics and analgesics such as opioids. For example, μ opioid receptor (MOR) agonists often modulate arousal, motor output, and autonomic tone and its cerebrovascular effects are inherently confounded under anesthesia or head fixation, highlighting the needs of imaging their *in vivo* actions in freely moving, awake animals. Given these advantages, freely moving fULM is particularly well suited for studying the *in vivo* actions of opioid analgesics. As a proof of principle, we chose to test our platform to image the *in vivo* action of SR-17018, an experimental G-protein biased MOR agonist that showed superior safety in animal models^28,29^.

**Figure 6:**
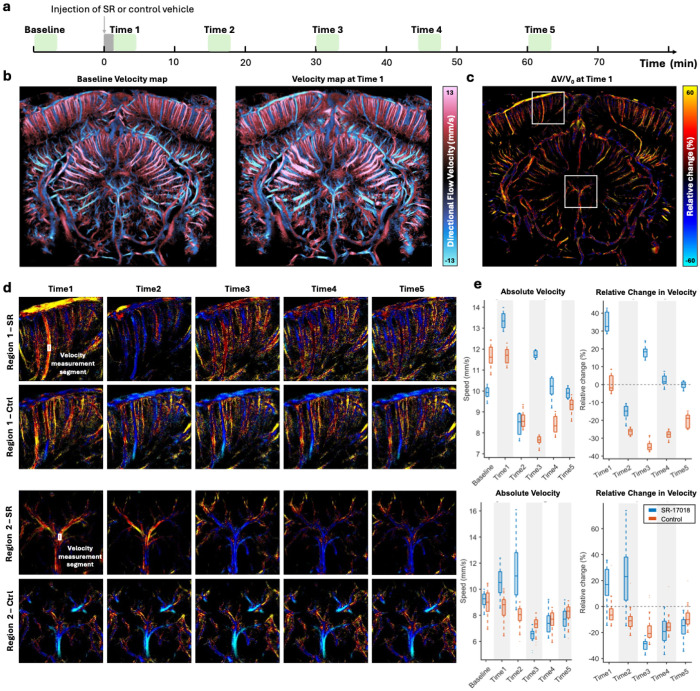
Single-vessel flow-velocity mapping reveals region-specific cerebrovascular responses to SR-17018 in a freely moving rat. **a,** Experimental timeline for fULM imaging before and after administration of SR-17018 or vehicle control. Baseline data were acquired before injection, followed by five post-injection imaging sessions spanning 60 min. **b,** Representative ULM directional flow-velocity maps at baseline and at the first post-injection time point after SR-17018 injection. **c,** Relative flow-velocity change (ΔV/V_0_) at Time 1, showing heterogeneous vascular responses after SR-17018 injection. White boxes indicate the two regions selected for further analysis. **d,** Enlarged ΔV/V_0_ maps from the two ROIs across all five post-injection time points for SR-17018 and control sessions. Region 1 corresponds to a cortical area containing pial and penetrating vessels, and Region 2 corresponds to the periaqueductal gray (PAG). In each region, a representative vessel segment used for quantitative analysis is indicated. **e,** Box plots summarizing absolute flow velocity and relative velocity change measured from the selected vessel segments in the two regions.

Serial fULM imaging was performed in a freely moving rat before and at five time points after administration of SR-17018, with vehicle-only sessions serving as controls (Fig. 6a). Fig. 6b shows ULM velocity maps acquired at baseline and immediately after SR-17018 injection, at a coronal plane (bregma −6.5 mm) including the periaqueductal gray (PAG), a primary center for opioid-mediated pain regulation and receptor adaptation^30^. To remove positional shift among imaging sessions, we registered all ULM images to a common baseline-derived template through the non-rigid motion-correction framework described above, which achieves pixel-level vessel alignment across time points to facilitate single-vessel-level comparison. To address the microbubble concentration variability issue during imaging, we used relative flow-velocity changes (ΔV/V_0_) rather than count-dependent parameters to yield a metric that is inherently insensitive to concentration fluctuations. This method allowed us to distinguish the changes of physiological and pharmacological origins. To validate this approach, we applied the same analysis to the stimulus-evoked dataset (Supplementary Figure 2).

With cross-session alignment established, ΔV/V_0_ maps computed at the first post-injection time point captured drug-induced flow-velocity changes at single-vessel resolution (Fig. 6c). In vessels that dilated following SR-17018 injection, elevated ΔV/V_0_ values appeared on both sides of the vessel wall, consistent with the diameter increases observed in Fig. 6b. To track the temporal evolution of these responses, regions of interest in the cortex and PAG were selected, and ΔV/V_0_ was monitored across all the post-injection sessions (Fig. 6d). Within the cortical ROI, pial vessels and their penetrating branches showed distinct hemodynamic responses to SR-17018 (Fig. 6d), which illustrates the spatial heterogeneity that only single-vessel resolution can capture. Time courses extracted from selected vessel segments (Fig. 6e) revealed region-dependent pharmacological dynamics. In a cortical penetrating vessel, SR-17018 produced an immediate velocity increase of approximately 30%, which dropped below baseline within 15 minutes and then gradually recovered. In contrast, PAG vessels showed a smaller (~20%) but more sustained increase, with flow velocity remaining elevated for over 15 minutes before gradually declining.

This sustained response observed in the PAG is consistent with biochemical evidence that SR-17018 maintains MOR signaling in this region, avoiding the rapid receptor desensitization associated with morphine^28^. Conversely, the transient cortical response may reflect the population-specific nature of opioid receptor regulation across the brain. In both regions, control sessions showed only a steady velocity decline, with no transient increase following injection. These results demonstrate that the fULM platform, combined with the cross-session processing framework, can resolve pharmacologically induced hemodynamic changes at single-vessel resolution in freely moving animals, providing a powerful approach for studying opioid action *in vivo*.

## Discussion

To our knowledge, this work presents the first validated platform for fULM in freely moving rats. Although fULM has emerged as a powerful neuroimaging modality capable of whole-brain functional imaging at single-vessel resolution, its use has been restricted to anesthetized or head-fixed preparations, which precludes application to naturalistic behavioral and pharmacological paradigms. Here, we remove this constraint by integrating dedicated experimental hardware with a motion-robust computational pipeline, enabling super-resolution hemodynamic imaging during unconstrained behavior. We validate the platform through visually evoked single-vessel functional mapping and demonstrate its utility for pharmacological neuroimaging by resolving opioid-induced microvascular responses across the brain, where anesthesia or head fixation would introduce substantial confounds.

Extending fULM to freely moving conditions introduces variability at every level of the acquisition, from microbubble delivery and probe fixation to image reconstruction. Addressing these challenges required concurrent advances in both experimental hardware and data processing. For stable acquisition, we integrated a magnetic-attachment dual-clamp probe holder for repeatable probe fixation, chronic jugular vein catheterization for uninterrupted microbubble infusion, and a shared pulley-based counterweight system that manages the cable and infusion line with minimal head loading. These hardware solutions suppress motion-induced degradation. The residual internal brain tissue displacement is addressed computationally through a coordinate-level motion correction strategy, in which non-rigid deformation fields estimated over ensemble windows are applied directly to localized microbubble positions before re-tracking, preserving velocity accuracy at the single-vessel level. For longitudinal and pharmacological experiments, cross-session image registration achieves pixel-level vessel alignment across time points, and a velocity-based ΔV/V_0_ metric decouples functional signals from the microbubble concentration fluctuations inherent to freely moving infusion conditions. Collectively, these hardware and computational advances provide the methodological basis for quantitative single-vessel functional analysis in freely moving animals.

Compared to head-fixed awake fULM, the primary advantage of the freely moving configuration is its direct compatibility with naturalistic behavioral paradigms. Although head-fixed imaging avoids the confounding effects of anesthesia, it is still inherently incompatible with behaviors that require unrestricted locomotion. In contrast, the freely moving preparation more closely reflects the animal’s native physiological state and can be directly combined with tasks involving exploration, fear conditioning, social interaction, or drug self-administration. The main trade-off is reduced transducer flexibility. Specifically, head-fixed platforms can support larger matrix arrays or row-column-addressed arrays for volumetric acquisition^31–33^, whereas freely moving implementations are currently limited to lightweight one-dimensional linear arrays, restricting imaging to a single plane.

The current platform is subject to several limitations that should be explicitly acknowledged. First, microbubble concentration stability remains a practical challenge. Within a single imaging session, elevated metabolic activity and movement-related perturbation of the infusion line may introduce slow fluctuations in circulating microbubble concentration. Although high-pass filtering was applied to mitigate this confounding factor, residual concentration-related modulation may still contribute to the measured functional signal. Across sessions, additional variability may arise from manual microbubble preparation and uncertainty associated with catheter dead volume, which may reduce inter-session comparability. Second, the current motion correction framework operates on 10-second ensemble windows and is therefore effective primarily for slow tissue drift. In contrast, rapid motion occurring within the ensemble window, particularly during active locomotion, is not directly corrected and may still introduce localization error. Third, the present implementation provides two-dimensional cross-sectional imaging only, which limits the completeness of whole-brain functional mapping and does not capture out-of-plane vascular dynamics. These limitations do not undermine the feasibility demonstrated here, but they do define the current technical boundaries of the platform and should be considered when interpreting the results.

The platform established in this study opens several important directions for future work. The most immediate application will be the integration of freely moving fULM with behavioral neuroscience paradigms in which natural locomotion is essential (e.g., social interaction, fear conditioning, drug addiction, and sleep-wake studies). On the technical side, improving microbubble delivery stability remains a high priority. Specifically, closed-loop infusion strategies that adjust the injection rate based on real-time *in vivo* concentration estimates, or alternatively the development of longer-circulating contrast agents, may mitigate concentration drift at its source, resulting in improved functional sensitivity and better inter-session consistency. Further advances in motion correction are also required. Shorter correction windows, or motion models capable of capturing sub-ensemble tissue displacement, would likely extend the utility of the platform to more active behavioral conditions.

Furthermore, the results of the SR-17018 study demonstrate that free-moving fULM can serve as a powerful platform for uncovering the spatiotemporal dynamics of drug action *in vivo*. By enabling brain-wide mapping of microvascular responses in animals under natural behavioral conditions, this approach allows differentiation between direct target engagement and secondary systemic effects that are often confounded under anesthesia or head-fixed conditions, such as those arising from respiratory or cardiovascular perturbations. In the case of SR-17018, the sustained hemodynamic activity observed in the PAG, a key region for pain modulation, highlights the ability of this platform to resolve region-specific and time-dependent opioid responses. More broadly, these findings illustrate how free-moving fULM can be applied to characterize pharmacological dynamics across diverse classes of therapeutic agents. This capability further underscores its potential for preclinical drug evaluation and for identifying longitudinal biomarkers of drug efficacy and tolerability.

Looking further ahead, three-dimensional volumetric fULM in freely moving animals represents a highly compelling but technically demanding goal. Achieving this goal will require sufficiently lightweight volumetric transducers with adequate volume rate, as well as corresponding solutions for data transmission bandwidth, tether design, and wearable hardware integration. If these technical barriers can be overcome, freely moving volumetric fULM may provide an unprecedented tool for large-scale functional microvascular imaging during natural behavior.

## Methods

### Animal preparation and usage overview

This study used four female Sprague-Dawley rats (8–12 weeks old) obtained from Charles River Laboratories. All procedures were approved by the Duke University Institutional Animal Care and Use Committee (IACUC Protocol #A028-25-04). Rats were maintained on a 12-hour light/dark cycle with *ad libitum* access to food and water. Animals were group-housed before cranial window surgery and transferred to individual housing afterward.

### Jugular vein catheterization and implantation of a vascular access button

For chronic intravenous access, rats underwent right jugular vein catheterization with implantation of a subcutaneous vascular access button (VAB). Anesthesia was induced with isoflurane (3%) and maintained at 1.0-1.5% throughout the procedure. Subcutaneous administration of Carprofen (5-10 mg/kg body weight) was provided to the animals for immediate pre-operative pain management. After aseptic preparation, the animal was placed supine on a heated surgical platform. A small incision was made over the right ventral neck, just rostral to the clavicle, and the underlying soft tissue was bluntly dissected to expose the right jugular vein. A short segment of the vein was isolated from the surrounding connective tissue, and two 6-0 silk sutures were placed beneath it. The rostral suture was tied to occlude the vessel, whereas the caudal suture was used to provide temporary control during catheter insertion. A small venotomy was then created between the two sutures, and a saline-filled polyurethane catheter (Instech Laboratories, Inc., C20PU-MJV2011) was inserted into the vein and advanced centrally toward the superior vena cava/right atrial junction. Proper catheter placement was confirmed by free aspiration of venous blood, followed by flushing to ensure unobstructed flow. The catheter was then secured to the vein with the pre-positioned sutures and tunneled subcutaneously to the interscapular region. A second incision was made between the scapulae, where a subcutaneous pocket was created for implantation of the VAB (Instech Laboratories, Inc., VABM1B/25). The catheter was passed through the tunnel, trimmed to the appropriate length, and connected to the button, with sufficient slack left to accommodate normal movement after recovery. The port was seated within the interscapular pocket, and catheter patency was re-confirmed through the implanted access button. At the completion of the procedure, the catheter was filled with sterile heparinized saline (20 IU/ml) to maintain patency, and both incisions were closed with sutures. During postoperative recovery and throughout the experimental period, the implanted line was flushed every 3 days with heparinized saline to preserve stable venous access for repeated administration of microbubbles or pharmacological agents.

### Cranial window surgery

The jugular vein catheterization and cranial window surgery were performed sequentially under a single anesthetic session on the same day. The surgical procedure was performed as previously described^14,34^. To prevent brain swelling, dexamethasone (0.5 mg/kg) was administered intraperitoneally before the cranial window surgery. With the animal secured in a stereotaxic frame, the scalp was incised and the temporalis muscle was detached from the bone surface using forceps. Tissue adhesive (3M, 70200742529) was used to hold back the retracted muscle and wound edges. A head plate was bonded to the skull using tissue adhesive together with dental cement (Sun Medical Co., Ltd, Super-Bond C&B). The skull was then gradually thinned with a high-speed rotary micromotor (Foredom, K.1070) to form a cranial window approximately 8 mm wide in the mediolateral direction, spanning from bregma −4mm to bregma −7mm. Thinning proceeded until the bone segment loosened sufficiently to be lifted away from the dura mater with forceps. A polymethyl pentene film (Goodfellow, ME311051) was placed directly over the craniotomy and affixed to the adjacent bone with tissue adhesive and dental cement. A layer of biocompatible silicone rubber (Smooth-On, Body Double-Fast Set) was applied over the window as a protective covering. Once the silicone had cured, anesthesia was discontinued, and the animal was allowed to recover.

### Post-surgical care, handling and habituation

Following surgery, each animal was returned to its home cage and placed on a heating pad to support thermoregulation throughout the recovery phase, until sternal recumbency was confirmed. Animals were subsequently monitored daily during the post-operative period. Post-operative care included intraperitoneal injection of dexamethasone (0.5 mg/kg) and subcutaneous injection of Carprofen (5–10 mg/kg) daily for three days to manage inflammation and surgical pain, respectively. Additional doses of Carprofen were administered as needed based on observed signs of discomfort. Heparin flushes (20 IU/ml) of the jugular catheter were also performed at least every 3 days to maintain patency. Beginning on post-operative day 7, animals underwent daily handling sessions in which the experimenter held and gently manipulated each rat to reduce anxiety and familiarize the animal with human contact. After three days of handling, habituation to the imaging setup commenced on day 10, during which animals wore the ultrasound transducer on the pulley-tethered platform. Session duration was progressively increased from approximately 10 minutes on the first day to up to 1 hour over the course of three days^34^. Counterbalance effectiveness was monitored throughout to ensure minimal head loading. If signs of distress such as excessive movement or vocalization were observed during any session, habituation was immediately discontinued.

### Preparation of SR-17018

SR-17018 stock solution was prepared by dissolving 5 mg SR-17018 in 500 μL sterile DMSO (Sigma-Aldrich, D8418) to yield a final concentration of 10 mg/mL. After vortexing to complete dissolution, the solution was sterile-filtered through a 0.22 μm membrane, aliquoted into 100 μL fractions, and stored at −20 °C protected from light. On the day of the experiment, a fresh 1 mg/mL working solution was prepared by diluting the stock 1:10 in a vehicle consisting of Tween-80 (Sigma-Aldrich, P4780) in saline. Specifically, 1 mL Tween-80 was first mixed with 8 mL sterile saline to prepare the dilution vehicle, after which 100 μL of the stock solution was added to 900 μL of this vehicle and vortexed until clear. The corresponding control vehicle was prepared in the same manner but without SR-17018, using an equivalent volume of DMSO diluted 1:10 in the same Tween-80/saline vehicle. The resulting solution was used immediately for subcutaneous administration at a dose of 6 mg/kg.

### Ultrasound imaging sequence

A Vantage NXT 256 system (Verasonics Inc., Kirkland, WA) was used for this experiment. The ultrasound system was connected to a linear-array transducer (LA15/128, Vermon SA.). Ultrasound was transmitted at a center frequency of 15.625 MHz. A 5-angle compounded plane wave technique was used (angles: −4°, −2°, 0°, 2°, and 4°), with a post-compounded frame rate of 1000 Hz. The ensemble size of each dataset was 800 frames, and the acquired radiofrequency (RF) data were saved for offline reconstruction. Beamforming was conducted using the Verasonics built-in program to reconstruct the in-phase and quadrature (IQ) data. The IQ data had a pixel size of half the wavelength in the axial direction and one wavelength in the lateral direction.

### SR-17018 Administration and Serial ULM Imaging

SR-17018 or the corresponding control vehicle was administered by subcutaneous injection. Prior to the experiment, each animal was allowed to freely move in the cage for environmental acclimation. The jugular vein catheter was then connected to the vascular access button mounted on the animal’s back. For baseline imaging, a syringe containing microbubble solution was connected to an infusion pump, and microbubbles were infused intravenously at a rate of 40 μL/min. Baseline ULM data were acquired immediately thereafter. After baseline acquisition, the catheter was disconnected, the ultrasound probe was removed, and the animal was taken out of the cage. One experimenter manually restrained the rat, while a second experimenter administered either SR-17018 or the control vehicle by subcutaneous injection. The animal was then returned to the cage, the probe was reattached, and the catheter was reconnected to the vascular access button. ULM imaging was then resumed following the same microbubble injection and imaging procedure as used for baseline acquisition. The first dataset acquired after drug administration was defined as the immediate post-injection time point (0 min). Subsequent imaging was performed at 15, 30, 45, and 60 min after drug administration. At each time point, fresh microbubble solution was prepared and injected to maintain more consistent intravascular microbubble concentration under matched injection conditions, thereby improving the comparability of ULM images across time points.

### Visual stimulation and fULM data acquisition

For visual stimulation experiments, animals were imaged in a dark environment while a white-light LED strip surrounding the cage delivered patterned visual stimulation. The stimulus consisted of flashing white light at 3 Hz for 20 s, followed by a 40 s resting period without stimulation. This 60-s cycle was repeated 5 times, resulting in a total stimulation experiment duration of 5 min. The LED strip was powered and controlled by an Arduino-based system. To ensure synchronization between stimulation delivery and ultrasound acquisition, the Arduino received trigger signals from the Verasonics system during imaging. The ultrasound acquisition procedure for the visual stimulation experiments followed the same workflow as that used in the SR-17018 study, except that no pharmacological intervention was performed. Throughout the entire 5-min recording, microbubbles were continuously infused through the chronic jugular catheter at a rate of 40 μL/min to maintain intravascular contrast for fULM imaging.

### ULM image processing

The IQ data were high-pass filtered at a 30 Hz cutoff to improve microbubble detection sensitivity. Based on the sign of the Doppler frequency shift, MBs were separated into two datasets corresponding to upward and downward flow directions relative to the transducer^35^. A clutter filter was applied to the IQ data using singular value decomposition (SVD)^36,37^, with the singular value threshold determined adaptively^38^. Following clutter removal, the MB data were spline-interpolated onto a finer grid with lateral and axial pixel dimensions equal to one-tenth of the acoustic wavelength (10 μm). On each interpolated frame, a 2D normalized cross-correlation was computed against an empirically measured point spread function of the imaging system, and the resulting correlation map was searched for regional maxima to identify MB centroid positions^39^. An intensity threshold was imposed to discard low-amplitude detections and minimize false localizations due to noise. Frame-by-frame centroid coordinates were then linked into trajectories using the uTrack algorithm^40,41^. Tracks shorter than 5 consecutive frames were discarded as unreliable. Two critical parameters governed the tracking: the maximum linking distance and the gap-closing distance, both set to 10 pixels (100 μm). Under these settings, only centroids separated by no more than 10 pixels in successive frames were assigned to the same trajectory, and track segments whose endpoints fell within this distance were merged into a single continuous path. Once all valid trajectories were established, their spatial accumulation produced a flow intensity map whose pixel values were square-root compressed to reduce dynamic range for visualization. Blood flow velocity was simultaneously derived by computing the inter-frame displacement along each trajectory; averaging the per-track velocities across all overlapping tracks then yielded a composite velocity map.

### Motion estimation and registration

To address the substantial motion encountered in freely moving imaging while preserving sufficient vascular information for registration, motion fields were estimated from temporally accumulated ULM images rather than from individual 1-s datasets. Specifically, a running temporal window of 10 seconds was used to construct partially accumulated ULM images, and the window was advanced with a step size of 1 second. The choice of the 10-s window represented a practical compromise. A shorter window would not accumulate a sufficient number of microbubbles to support reliable vascular registration, whereas a longer window would allow excessive within-window motion that could no longer be reasonably approximated by a single motion field. Although each 10-s accumulation was not fully saturated in terms of vascular reconstruction, it preserved sufficient microvascular structural information to support image registration while maintaining a relatively dense temporal sampling of motion, resulting in a motion field estimate at every second throughout the acquisition. Each accumulated ULM image was saved and subsequently registered using ANTsPy^26,27^, a widely used image registration toolkit. In the present implementation, only the deformable registration stage was performed, whereas all affine-related stages were omitted. Accordingly, a symmetric normalization (SyN) deformable transformation was directly estimated between each moving image and a fixed reference image without rigid or affine pre-alignment.

For each registration, the fixed image was defined as the first accumulated ULM image in the sequence. Deformable registration was then performed using the cross-correlation metric with a sampling radius of 4. A four-level multi-resolution optimization scheme was used with SyN iteration numbers of 100, 70, 50, and 20. The gradient step size was set to 0.15, while Gaussian smoothing was applied to the update field and the total deformation field with a sigma of 3. In ANTsPy, these parameters control the smoothness of the incremental displacement updates and the accumulated transform field during optimization, facilitating stable deformable registration.

The forward transform produced by each registration was extracted from the ANTsPy output and saved as a dense deformation field. This procedure yielded a time-resolved sequence of dense motion fields, each describing the nonrigid displacement between a given 10-s accumulated ULM image and the fixed reference image. After motion field estimation, the displacement field was applied to the localized microbubble coordinates rather than directly to the final ULM rendering. The motion-corrected microbubble coordinates were then used for subsequent tracking and ULM reconstruction. This coordinate-level correction ensured that inter-frame microbubble displacement was quantified more accurately, thereby improving the fidelity of downstream measurements derived from bubble trajectories, including track length and flow velocity.

### Relative flow-velocity analysis and confidence-weighted display

For cross-session pharmacological imaging, direct comparison of microbubble-count-based ULM measures is confounded by variability in circulating microbubble concentration across imaging sessions. To address this issue, drug-induced hemodynamic changes were quantified using relative flow-velocity maps rather than count-dependent parameters. Specifically, a ULM velocity map was reconstructed for each imaging session, and the baseline velocity map acquired before drug administration was used as the reference map (V_0_). Relative flow-velocity changes were then computed pixel-wise as ΔV/V_0_ for each post-injection session. Because raw relative velocity maps were visually unstable in regions with sparse microbubble sampling, particularly in small vessels, the underlying velocity maps were first spatially smoothed with a Gaussian filter (σ = 0.5) before calculation of the relative change maps. To improve the interpretability of the displayed ΔV/V_0_ maps, a confidence-weighted masking strategy was further applied based on the corresponding ULM microbubble-count map. The count map was first normalized to the range of 0 to 1 after square-root transformation (γ = 0.5) to enhance the visibility of small vessels. This normalized map was then used to define pixel opacity in the relative velocity display, such that regions with higher microbubble counts, and therefore more reliable velocity estimates, were shown with higher confidence, whereas poorly sampled regions were progressively suppressed. In the visualization shown in Supplementary Figure 2, two opacity-transfer schemes were evaluated. In mask 1, pixels with normalized ULM values greater than 0.15 were fully displayed, pixels below 0.01 were fully suppressed, and opacity was linearly ramped between these bounds. In mask 2, the corresponding lower and upper bounds were 0.05 and 0.30, respectively. This procedure was used to improve display quality and to visually down-weight regions where velocity estimates were less reliable because of limited microbubble sampling.

## Supplementary Material

This is a list of supplementary files associated with this preprint. Click to download.

• SupplementaryVideo1.mp4

• SupplementaryVideo2.mp4

• image7.png

• image8.png
